# Genetic background impacts the timing of synaptonemal complex breakdown in *Drosophila melanogaster*

**DOI:** 10.1007/s00412-020-00742-9

**Published:** 2020-10-17

**Authors:** Emily R. Wesley, R. Scott Hawley, Katherine Kretovich Billmyre

**Affiliations:** 1 Stowers Institute for Medical Research, Kansas City, MO 64110 USA; 2 University of Missouri-Kansas City, Kansas City, MO 64110 USA; 3 Department of Molecular and Integrative Physiology, University of Kansas Medical Center, Kansas City, KS 66160 USA

**Keywords:** Genetic background, Meiosis, Synaptonemal complex

## Abstract

Experiments performed in different genetic backgrounds occasionally exhibit failure in experimental reproducibility. This is a serious issue in *Drosophila* where there are no standard control stocks. Here, we illustrate the importance of controlling genetic background by showing that the timing of a major meiotic event, the breakdown of the synaptonemal complex (SC), varies in different genetic backgrounds. We assessed SC breakdown in three different control stocks and found that in one control stock, *y w; sv*^*spa-pol*^, the SC broke down earlier than in *Oregon-R* and *w*^*1118*^ stocks. We further examined SC breakdown in these three control backgrounds with flies heterozygous for a null mutation in *c(3)G*, which encodes a key structural component of the SC. Flies heterozygous for *c(3)G* displayed differences in the timing of SC breakdown in different control backgrounds, providing evidence of a sensitizing effect of this mutation. These observations suggest that SC maintenance is associated with the dosage of *c(3)G* in some backgrounds. Lastly, chromosome segregation was not affected by premature SC breakdown in mid-prophase, consistent with previous findings that chromosome segregation is not dependent on full-length SC in mid-prophase. Thus, genetic background is an important variable to consider with respect to SC behavior during *Drosophila* meiosis.

**Electronic supplementary material:**

The online version of this article (10.1007/s00412-020-00742-9) contains supplementary material, which is available to authorized users.

## Introduction

A cornerstone of genetics research is the assumption that phenotype is directly influenced by genotype. Researchers seek to minimize genetic noise and confounding variables by holding all variables constant. While researchers will often manipulate environmental variables to screen for phenotypic effects, the importance of genetic background is commonly overlooked. New mutants are rarely examined in different wild-type genetic backgrounds.

Nonetheless, genetic differences between background strains can have a tremendous impact on the reproducibility of experiments (Chandler et al. 2013). This is especially problematic in fields such as *Drosophila* research, where many different control stocks exist and there is no community standard for “wild type.” Furthermore, some studies fail to specify which stock was used as a control, disregarding the importance of genetic background. The decision to use one control over another is often based on experimental design or convenience, resulting in a wide variation in controls among fly laboratories.

In contrast, this concern is addressed in other organisms, such as *C. elegans*, yeast, and mice, by using a standard set of wild-type control stocks in the majority of laboratories (Mortimer and Johnston 1986; Sterken et al. 2015; Sarsani et al. 2019). New mutations are either created directly in these stocks or introgressed into them prior to detailed analysis. Comparisons of a given mutant’s phenotype in these well-characterized backgrounds have been highly informative. Further, work in yeast has revealed the complexity of interaction between mutations, genetic variation, and the environment (Dowell et al. 2010; Mullis et al. 2018). A common example is the difference in sporulation efficiency between two *S. cerevisiae* strains, SK1 and S288c; SK1 is highly efficient at sporulation while S288c is not (Keeney 2009). Lastly, the mouse community has long been interested in the influence of genetic variation on phenotype and has made great strides in showing the necessity of examining phenotypes in multiple genetic backgrounds (Doetschman 2009; Justice and Dhillon 2016; Sittig et al. 2016).

On the other hand, there are numerous examples in *Drosophila* in which a mutant exhibited different phenotypes in different genetic backgrounds. For example, the *Indy* mutant lengthens lifespan in *Drosophila* (Rogina et al. 2000). However, further examination in other genetic backgrounds revealed that this phenotype was likely due to genetic background effects, rather than a specific mutation (Toivonen et al. 2007). Similarly, the *scalloped (E3)* mutation displays markedly different phenotypes in the wild-type stocks Samarkand and Oregon-R (Dworkin et al. 2009). Genetic background comparison can also be used as a tool to learn more about the function of a gene. For example, comparing the phenotype of *mushroom body miniature* mutants in different genetic backgrounds allowed the elucidation of two different roles for the gene: brain anatomy and associated olfactory learning (De Belle and Heisenberg 1996). Unfortunately, such comparisons are by no means standard in *Drosophila* genetics.

**Fig. 1:**
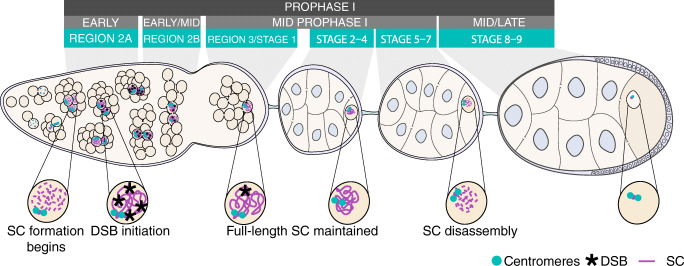
Model of oocyte development in the *Drosophila* ovariole. SC (purple) formation begins in multiple nuclei during early prophase (region 2A). DSBs (black) are formed and some are resolved into crossovers during early to mid-prophase (region 2A to stage 1). By mid-prophase (stage 1), one cell is designated as the oocyte while the surrounding cells become nurse cells. Full-length SC persists into mid prophase (stage 1 through stage 5) and then begins disassembly from the chromosome arms in mid to late prophase (stage 5–7)

Genetic background appears to be more important for phenotypes that cause intermediate effects than it is for those phenotypes that have either weak or severe effects (Chandler et al. 2017). We focus here on the timing of synaptonemal complex (SC) breakdown, a variable phenotype of interest to our laboratory. The SC is a large tripartite protein structure formed between homologous chromosomes in meiotic prophase. It is required to both maintain chromosome pairing and for the formation of crossovers (reviewed in Cahoon and Hawley 2016). The SC is a highly conserved structure that is composed of lateral and central elements with a central region containing a transverse filament protein that connects the two homologous chromosomes. In *Drosophila*, the central region of the SC contains two proteins, Corona (Cona) and Corolla, in addition to the transverse filament protein, C(3)G (Page and Hawley 2001; Page et al. 2008; Collins et al. 2014). If any component of the central region is absent, the SC does not form, which results in a lapse of chromosome pairing and a complete loss of genetic exchange. The absence of exchange causes high levels of chromosome missegregation at meiosis I (Page and Hawley 2001; Page et al. 2008; Collins et al. 2014). The SC is fully assembled by early prophase (region 2A; Fig. 1). The SC remains fully assembled throughout early prophase and most of mid-prophase (stages 1–6) during which DSBs are induced and designated as either non-crossovers or crossovers (Page and Hawley 2001).

In *Drosophila*, SC disassembly is said to begin at the end of mid-prophase (stages 5–7; Fig. 1) (Page and Hawley 2001; Takeo et al. 2011) and conclude by mid/late prophase (stage 7–9; Fig. 1). The impact of genetic variation on the timing of SC breakdown has not been examined, and few studies exist in which mutants exhibit premature SC breakdown (Webber et al. 2004; Billmyre et al. 2019). Furthermore, the mutants in those studies were compared to genetically similar backgrounds to minimize genetic diversity. Historically, it has been impossible to study SC breakdown/disassembly because a mutation in any central region gene causes a complete loss of the SC. Therefore, little is known about the mechanisms that regulate SC maintenance and disassembly.

Here we report that genetic background of *Drosophila melanogaster* affects the timing of SC breakdown. The three wild-type stocks examined were *y w; sv*^*spa-pol*^, *Oregon-R* (*OreR*), and *w*^*1118*^, which have been previously used as controls in many studies examining meiotic processes (Page and Hawley 2001; Resnick et al. 2009; Takeo et al. 2011; Hughes et al. 2019; Billmyre et al. 2019). We found that the *y w; sv*^*spa-pol*^ stock exhibited SC breakdown significantly earlier than *OreR* and *w*^*1118*^. Additionally, flies that are heterozygous for a null allele of *c(3)G* displayed even earlier breakdown of the SC in the *y w; sv*^*spa-pol*^ background, revealing a sensitizing effect of heterozygosity for *c(3)G*. Surprisingly, the *y w; sv*^*spa-pol*^ background acted as a *c(3)G*-specific dominant enhancer, as flies heterozygous for a *corolla* null allele did not exhibit the premature SC breakdown phenotype. This work highlights the complex role that genetic background can have on meiotic processes in *Drosophila*, supports previous work suggesting that background can affect reproducibility, and is evidence of a dosage effect of *c(3)G* in certain backgrounds.

## Results

### Variation occurs in the timing of SC breakdown in *Drosophila* control stocks

SC assembly and maintenance is a highly regulated process that ensures the proper alignment of chromosomes for successful recombination and segregation (reviewed in Cahoon and Hawley 2016). Failing to assemble full-length SC or premature disassembly of the SC results in recombination and pairing defects (Page and Hawley 2001; Manheim and McKim 2003; Webber et al. 2004; Page et al. 2008; Collins et al. 2014; Billmyre et al. 2019). It is currently unknown what regulates either assembly (See Hughes et al. 2019), maintenance, or disassembly of the SC.

**Fig. 2:**
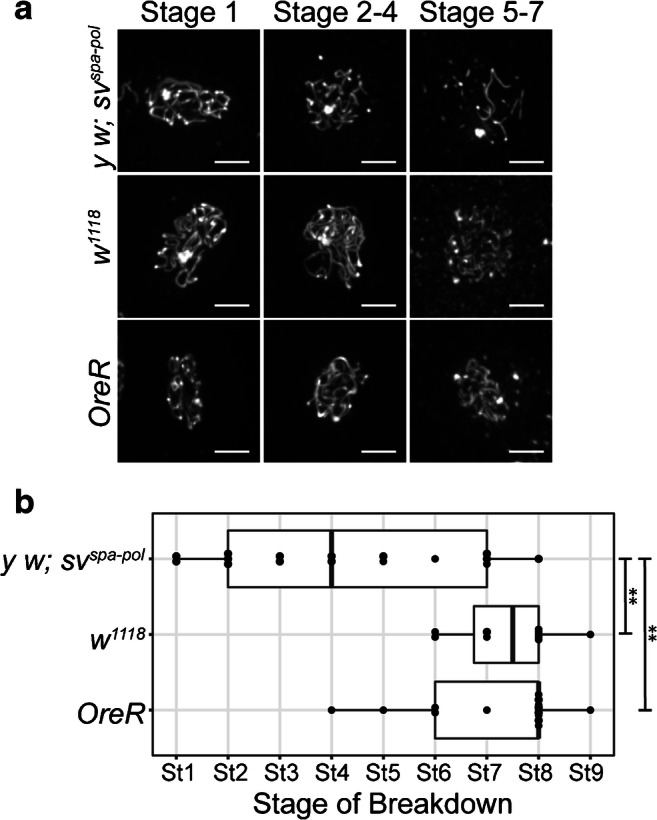
Timing of SC breakdown in *Drosophila* control stocks. **a** Max projections of representative oocytes stained with an antibody against Corolla in the control stocks *y w; sv*^*spa-pol*^, *OreR*, and *w*^*1118*^ throughout mid-prophase (stage 1–7) (Scale bars, 2 μm). The SC is fragmented in *y w; sv*^*spa-pol*^ during stage 5–7. A haze of Corolla is normally present as the SC is breaking down. **b** Quantification of the stage of SC breakdown in the control stocks *y w; sv*^*spa-pol*^, *OreR*, and *w*^*1118*^ from mid-prophase (stage 1) to mid-/late prophase (stage 8–9). ** p< 0.001 by Mann–Whitney *U* test. *y w; sv*^*spa-pol*^: *n* = 17; *OreR*: *n* = 16; *w*^*1118*^: *n* = 16

To explore the role that genetic background may play in the regulation of SC breakdown in *Drosophila* females, we asked whether timing of SC breakdown was consistent among the three control stocks commonly used for examining meiotic mutants. These control stocks (*y w; sv*^*spa-pol*^, *OreR*, and *w*^*1118*^) were stained with an antibody recognizing the central element protein, Corolla, to determine the median stage of euchromatic SC breakdown (See methods, Fig. S1). The timing of SC breakdown was assessed based on egg chamber morphology as previously described (Spradling 1993). Because mid-prophase extends throughout many stages of development (stage 1–7), in this paper, we will reference the developmental time point using the more specific term of stage numbers (Fig. 1). The median stage of SC breakdown observed in *y w; sv*^*spa-pol*^ was stage 4; however, the stage of SC breakdown varied widely in *y w; sv*^*spa-pol*^ flies from stage 1 to stage 8 (Fig. 2). This was significantly different than the median stage of breakdown observed in *OreR* females (Fig. 2: stage 8, Range: stage 4–9, *p* = 0.0007) and *w*^*1118*^ (Fig. 2: stage 8, Range: stage 6–9, *p* = 0.0004). The ranges of SC breakdown seen in *OreR* and *w*^*1118*^ stocks are consistent with previously published results (Page and Hawley 2001; Takeo et al. 2011). The difference in SC breakdown observed between the *y w; sv*^*spa-pol*^, *OreR,* and *w*^*1118*^ control stocks suggests a possible relationship between genetic background and the timing of SC breakdown.

### *c(3)G* heterozygotes displayed premature SC breakdown in the *y w; sv*^*spa-pol*^ control background

Next, we investigated whether heterozygotes carrying a single copy of a null allele of *c(3)G* would exacerbate the SC breakdown phenotype observed in the *y w; sv*^*spa-pol*^ background. The *c(3)G* gene encodes a transverse filament protein that spans the width of the SC and is essential for SC formation (Page and Hawley 2001). Heterozygosity for a *c(3)G* null allele might be expected to decrease the level of C(3)G protein. We reasoned that reducing the dosage of *c(3)G* may exacerbate the effect on SC breakdown observed in the *y w; sv*^*spa-pol*^ background. We examined SC disassembly in the presence of heterozygosity for either of two null alleles of *c(3)G* (*c(3)G*^*1*^ and *c(3)G*^*68*^). Specifically, *c(3)G*^*68*^ contains a truncating SNP while *c(3)G*^*1*^ has a spontaneous insertional mutation (Page and Hawley 2001). Previous work with *c(3)G*^*68*^ heterozygotes found no changes in recombination compared to wild-type controls (Miller 2020). However, *c(3)G*^*1*^ heterozygotes have been reported to exhibit increased recombination in some genetic intervals (Hinton 1966; Meyer et al. 2014). It is also possible that this variation in recombination phenotype may be the effect of minor variations in genetic background: Hinton assessed *X* chromosome recombination using different multiply marked *X* chromosomes and showed that recombination was not consistently altered in *c(3)G*^*1*^ heterozygotes compared to controls when different *X* chromosomes were tested (Hinton 1966).

Both null alleles of *c(3)G* were used to create heterozygotes in each control background (*y w; sv*^*spa-pol*^, *OreR*, and *w*^*1118*^), to ensure that the phenotype was not due to a specific null allele of *c(3)G* (Crosses diagrammed in Fig. S2; full genotypes in Table 1 and methods). Due to the *c(3)G* heterozygotes only containing half the genetic material of the control stocks (see Fig. S2), we cannot directly compare SC breakdown in the *c(3)G* heterozygotes to the control stocks. Instead, we are limited to comparing SC breakdown in *c(3)G* heterozygotes introduced into different control backgrounds.

**Table 1:** Full genotypes of *c(3)G* and *corolla* heterozygotes

Genotype	Abbreviated genotype
*y w/y; +/+; th c(3)G*^*68*^*/+; sv*^*spa-pol*^*/+*	*y w; sv*^*spa-pol*^ *c(3)G*^*68*^ heterozygote
*y w/+; +/+; ru h th st c(3)G*^*1*^ *e*^*s*^ *ca/+; sv*^*spa-pol*^*/+*	*y w; sv*^*spa-pol*^ *c(3)G*^*1*^ heterozygote
*+(OreR)/y; +/+; th c(3)G*^*68*^*/+; +/+*	*OreR c(3)G*^*68*^ heterozygote
*+(OreR)/+; +/+; ru h th st c(3)G*^*1*^ *e*^*s*^ *ca/+; +/+*	*OreR c(3)G*^*1*^ heterozygote
*w*^*1118*^*/y; +/+; th c(3)G*^*68*^*/+; +/+*	*w*^*1118*^ *c(3)G*^*68*^ heterozygote
*w*^*1118*^*/+; +/+; ru h th st c(3)G*^*1*^ *e*^*s*^ *ca/+; +/+*	*w*^*1118*^ *c(3)G*^*1*^ heterozygote
*y w/y sc w*^*+*^ *cv v corolla*^*39*^*; +/+; +/+; sv*^*spa-pol*^*/+*	*y w; sv*^*spa-pol*^ *corolla*^*39*^ heterozygote

**Fig. 3:**
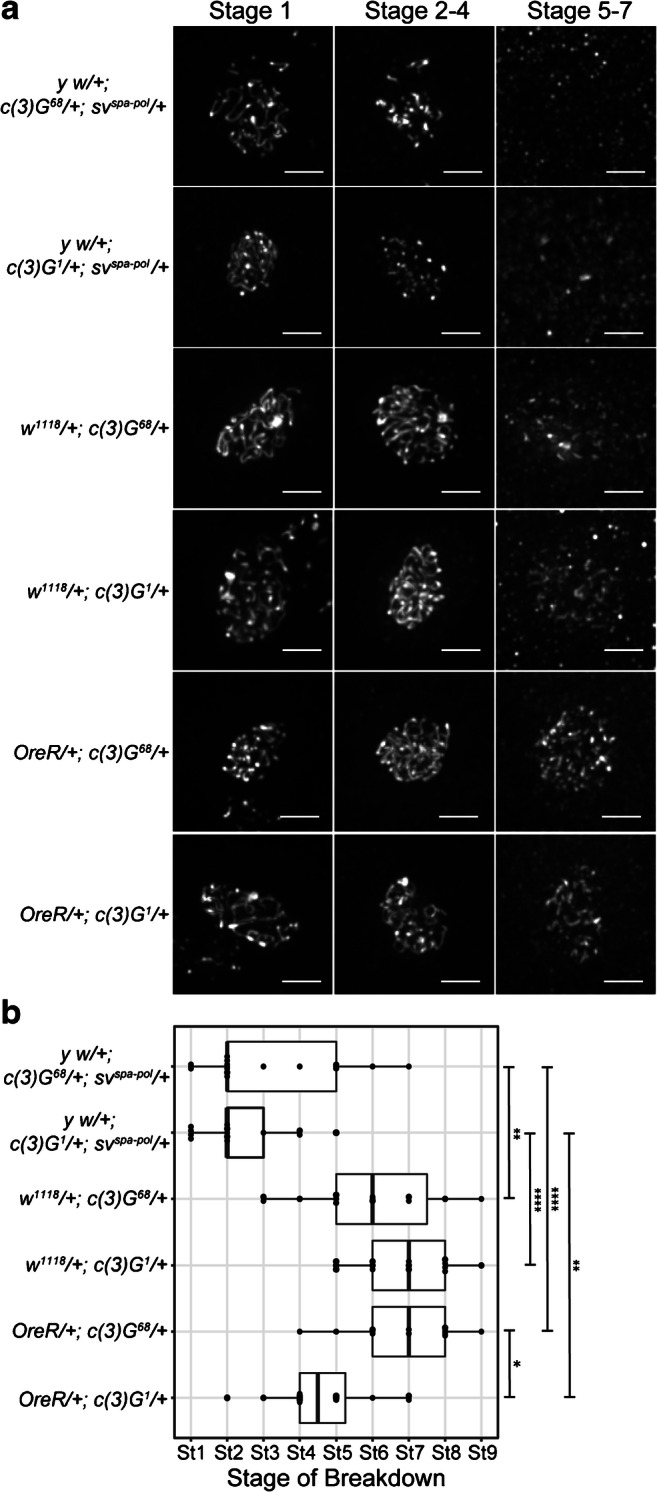
SC breakdown is accelerated in *c(3)G* heterozygotes in a *y w; sv*^*spa-pol*^ background. **a** Max projections of representative oocytes stained with an antibody against Corolla in *y w; sv*^*spa-pol*^ *c(3)G*^*1*^ and *c(3)G*^*68*^ heterozygotes, *OreR c(3)G*^*1*^ and *c(3)G*^*68*^ heterozygotes, and *w*^*1118*^ *c(3)G*^*1*^ and *c(3)G*^*68*^ heterozygotes throughout mid-prophase (stage 1–7) (Scale bars, 2 μm). The SC is fragmented in *y w; sv*^*spa-pol*^ *c(3)G*^*1*^ and *c(3)G*^*68*^ heterozygotes during stage 2–4. The SC is diffuse in *y w; sv*^*spa-pol*^ *c(3)G*^*1*^ and *c(3)G*^*68*^ heterozygotes during stage 5–7. The SC is punctate in *w*^*1118*^ *c(3)G*^*68*^ heterozygotes during stage 5–7. The SC is fragmented in *w*^*1118*^ *c(3)G*^*1*^, *OreR c(3)G*^*1*^ and *c(3)G*^*68*^ heterozygotes during stage 5–7. B) Quantification of the stage of SC breakdown in *y w; sv*^*spa-pol*^ *c(3)G*^*1*^ and *c(3)G*^*68*^ heterozygotes, *OreR c(3)G*^*1*^ and *c(3)G*^*68*^ heterozygotes, and *w*^*1118*^ *c(3)G*^*1*^ and *c(3)G*^*68*^ heterozygotes from mid-prophase (stage 1) to mid-/late prophase (stage 8–9). SC breakdown in *y w; sv*^*spa-pol*^ *c(3)G*^*68*^ heterozygotes is significantly earlier when compared to *w*^*1118*^ *c(3)G*^*1*^ heterozygotes (*p* < 0.0001) and *OreR c(3)G*^*1*^ heterozygotes (*p* = 0.014). However, the SC breaks down significantly later in *w*^*1118*^ *c(3)G*^*1*^ heterozygotes than in *OreR c(3)G*^*1*^ heterozygotes (*p* = 0.0003). In *y w; sv*^*spa-pol*^ *c(3)G*^*1*^ heterozygotes, the SC breaks down significantly earlier than in both *OreR c(3)G*^*68*^ and *w*^*1118*^ *c(3)G*^*68*^ heterozygotes (*p* < 0.00001). *p < 0.01, **p< 0.001, and ****p< 0.00001 by Mann–Whitney *U* test. *y w; sv*^*spa-pol*^ *c(3)G*^*1*^ heterozygote: *n* = 17; *y w; sv*^*spa-pol*^ *c(3)G*^*68*^ heterozygote: *n* = 17; *OreR c(3)G*^*1*^ heterozygote: *n* = 16; *OreR c(3)G*^*68*^ heterozygote: *n* = 16; *w*^*1118*^ *c(3)G*^*1*^ heterozygote: *n* = 17; *w*^*1118*^ *c(3)G*^*68*^ heterozygote: *n* = 15

The *y w; sv*^*spa-pol*^ background appears to expedite SC breakdown in heterozygotes for both *c(3)G* alleles tested (Fig. 3). SC breakdown in *y w; sv*^*spa-pol*^ *c(3)G* heterozygotes was significantly earlier when compared to *w*^*1118*^ and *OreR c(3)G* heterozygotes (Fig. 3). *y w; sv*^*spa-pol*^ *c(3)G*^*68*^ heterozygotes exhibited breakdown at stage 2, while SC in *w*^*1118*^ *c(3)G*^*68*^ and *OreR c(3)G*^*68*^ heterozygotes broke down significantly later at stage 6 and 7, respectively (Fig. 3, *p* = 0.0005, *p* < 0.00001). Similar trends were apparent in *c(3)G*^*1*^ heterozygotes. We observed accelerated SC breakdown in *y w; sv*^*spa-pol*^ *c(3)G*^*1*^ heterozygotes when compared to *w*^*1118*^ *c(3)G*^*1*^ heterozygotes (Fig. 3, *y w; sv*^*spa-pol*^ *c(3)G*^*1*^ hets: stage 2 versus *w*^*1118*^ *c(3)G*^*1*^ hets: stage 7, *p* < .00001). Although *OreR c(3)G*^*1*^ heterozygotes lost SC earlier than *OreR c(3)G*^*68*^ heterozygotes at stage 5 compared to stage 7 (Fig. 3, *p* = 0.002), breakdown in *OreR c(3)G*^*1*^ heterozygotes was not as early as in *y w; sv*^*spa-pol*^ *c(3)G*^*1*^ heterozygotes (Fig. 3, stage 5 versus stage 2, *p* = 0.0001).

Collectively, these observations reveal that *c(3)G* heterozygotes in a *y w; sv*^*spa-pol*^ background have significantly expedited SC breakdown when compared to *OreR* and *w*^*1118*^ *c(3)G* heterozygotes. This is similar to the trend observed when comparing SC breakdown in control stocks (*y w; sv*^*spa-pol*^, *OreR*, and *w*^*1118*^), and thus, the *y w; sv*^*spa-pol*^ background appears to expedite SC breakdown in both *c(3)G*^*+*^ and *c(3)G/+* heterozygotes (Figs. 2 and 3). The phenotypic similarity between the two *c(3)G* mutants strongly suggests the presence of a background modifier of C(3)G in the *y w; sv*^*spa-pol*^ stock, as the two mutants contain very different mutations within *c(3)G* and have been maintained in different backgrounds (Fig. S2).

### *corolla* heterozygotes do not have an SC breakdown defect in the *y w; sv*^*spa-pol*^ background

**Fig. 4:**
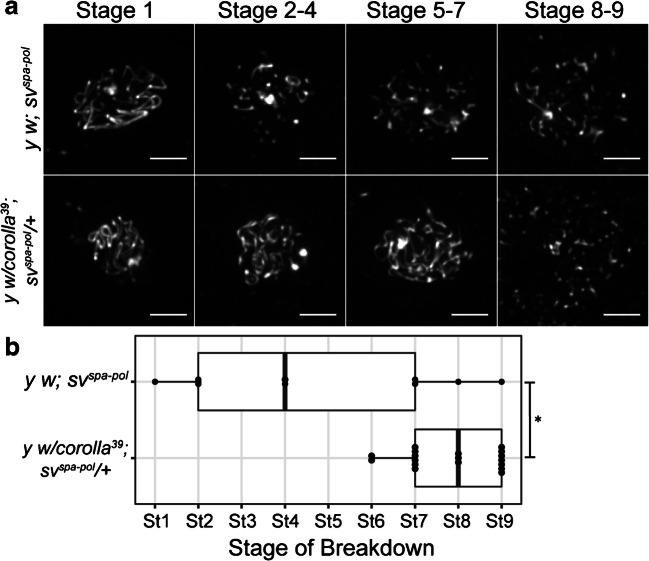
*corolla*^*39*^ heterozygotes do not display altered timing of breakdown. **a** Max projections of representative oocytes stained with an antibody against Corolla in *y w; sv*^*spa-pol*^ and *y w; sv*^*spa-pol*^ *corolla*^*39*^ heterozygotes from mid-prophase (stage 1–7) to mid-/late prophase (stage 8–9) (Scale bars, 2 μm). The SC is fragmented in *y w; sv*^*spa-pol*^ during stage 2–4, stage 5–7, and stage 8–9. The SC is fragmented in *y w; sv*^*spa-pol*^ *corolla*^*39*^ heterozygotes during stage 8–9. B) Quantification of stage of SC breakdown in the control stock *y w; sv*^*spa-pol*^ and *y w; sv*^*spa-pol*^ *corolla*^*39*^ heterozygotes from mid-prophase (stage 1) to mid-/late prophase (stage 8–9). *P< 0.01 by Mann–Whitney U test. *y w; sv*^*spa-pol*^: *n* = 10; *y w; sv*^*spa-pol*^ *corolla*^*39*^ heterozygote: *n* = 18. The median stage of breakdown in *y w; sv*^*spa-pol*^ *corolla*^*39*^ heterozygotes (stage 8) trends toward that seen in the *OreR* and *w*^*1118*^ control stocks (stage 8)

We next analyzed if the early SC breakdown in *c(3)G* heterozygotes in a *y w; sv*^*spa-pol*^ background was specific to *c(3)G* mutants, or might also be observed in flies heterozygous for mutations in genes encoding other SC components. If the *y w; sv*^*spa-pol*^ genetic background was affecting the entire SC structure, then perhaps heterozygosity for null alleles of genes encoding other SC components would affect SC perdurance in a similar fashion to *c(3)G* heterozygosity. As noted above, the *corolla* gene encodes another central region component of the SC. Heterozygotes for a null allele of *corolla* (*corolla*^*39*^) were examined in the *y w; sv*^*spa-pol*^ background by crossing *y w; sv*^*spa-pol*^ flies to a stock containing a *corolla*^*39*^ allele to produce *y w; sv*^*spa-pol*^ *corolla*^*39*^ heterozygotes (Cross diagramed in Fig. S2; full genotype in Table 1 and methods). During the assessment of the *y w; sv*^*spa-pol*^ *corolla*^*39*^ heterozygotes, we repeated an analysis of the *y w; sv*^*spa-pol*^ control stock in order to verify the median and range of the stage of breakdown. Premature SC breakdown was not observed in *corolla*^*39*^ heterozygotes in a *y w; sv*^*spa-pol*^ background (Fig. 4; stage 8, Range: stage 6–9). Indeed, SC breakdown was significantly later than the median stage of breakdown in the *y w; sv*^*spa-pol*^ control stock (stage 4, Range: stage 1–9, *p* = 0.008). The *y w; sv*^*spa-pol*^ *corolla*^*39*^ heterozygotes trended toward the wild-type timing of SC breakdown (Fig. 4) seen in *OreR* and *w*^*1118*^ control stocks (compare to Fig. 2). Additionally, the timing of SC breakdown in *y w; sv*^*spa-pol*^ *corolla*^*39*^ heterozygotes was significantly different when compared to the median stage of breakdown observed in *y w; sv*^*spa-pol*^ *c(3)G* heterozygotes (Fig. 3, stage 2, *p* < .00001). Our observations suggest that premature SC breakdown is not a general phenotype of flies heterozygous for mutations in SC genes, but rather it appears to be specific to *c(3)G* heterozygotes in a *y w; sv*^*spa-pol*^ background.

### The *y w; sv*^*spa-pol*^ control stock does not contain mutations in the coding sequence of *c(3)G*

One possible explanation for the different phenotypes in control backgrounds is that weak mutations in genes encoding SC proteins were present in *y w; sv*^*spa-pol*^ but not *OreR* and *w*^*1118*^ backgrounds. Such mutations could result in reduced levels of C(3)G and a greater acceleration in SC breakdown when one *c(3)G* null allele was present. Using Sanger sequencing, the *c(3)G* genes of the three control stocks (*y w; sv*^*spa-pol*^, *OreR*, and *w*^*1118*^) were compared to the reference genome (*ISO-1*) (Thurmond et al. 2018). No coding changes were identified in *c(3)G* when comparing the control stocks to each other (Table 2). However, 11 SNPs were present outside of the coding region of the *c(3)G* gene in the *OreR* and *w*^*1118*^ control stocks when compared to the reference genome and *y w; sv*^*spa-pol*^ sequence (Table 2). Seven of these SNPs were located within the 3’ UTR of *c(3)G*, two were located directly downstream of the gene, and two were located approximately 800 and 1100 base pairs upstream of *c(3)G*. Additionally, the presence of a mutation in a modifier or enhancer region outside of *c(3)G* in *y w; sv*^*spa-pol*^ flies cannot be ruled out.

**Table 2:** Differences between *c(3)G* in *ISO-1*, *y w; sv*^*spa-pol*^, *OreR,* and *w*^*1118*^

*ISO-1*	*y w; sv*^*spa-pol*^	*OreR and w*^*1118*^	Location
G	G	A	Downstream of gene
G	G	A	Downstream of gene
G	G	C	3’ UTR
A	A	T	3’ UTR
C	C	T	3’ UTR
AGATATG	AGATATG	Not present	3’ UTR
A	A	T	3’ UTR
G	G	C	3’ UTR
Not present	Not present	TAT	3’ UTR
G	G	A	800 bp upstream
A	A	G	1100 bp upstream

SNPs from 2015 bp upstream, 473 bp downstream, and within the *c(3)G* gene of *y w; sv*^*spa-pol*^ that differ from *c(3)G* in *OreR* and *w*^*1118*^. There are no coding changes in the *c(3)G* gene in *y w; sv*^*spa-pol*^ when compared to *OreR* and *w*^*1118*^. Sequencing results were generated using Sanger sequencing and primers listed in Key Resource Table. *ISO-1* sequence is from the reference sequence (FB2020_02) available on FlyBase (Thurmond et al. 2018)

Many germline genes are regulated by the 3’ UTR (Merritt et al. 2008), so it is possible that these SNPs are regulating SC breakdown. This has not been extensively studied in *Drosophila*. We used qPCR to test if *c(3)G* RNA levels differed in *y w; sv*^*spa-pol*^ ovaries compared to *OreR*, and *w*^*1118*^ ovaries in both *c(3)G*^*1*^ and *c(3)G*^*68*^ heterozygotes. Interestingly, *c(3)G* RNA expression in the *y w; sv*^*spa-pol*^ controls was less than half of that in *OreR*, and *w*^*1118*^ (Fig. S3), suggesting the *y w; sv*^*spa-pol*^ ovaries contained less *c(3)G* RNA. However, *c(3)G*^*1*^ heterozygotes in all control backgrounds had significantly less *c(3)G* expression than *OreR*, and *w*^*1118*^ control backgrounds (Fig. S3), even though *c(3)G*^*1*^ *OreR* and *w*^*1118*^ heterozygotes do not exhibit the same premature SC loss. This suggests that decreased *c(3)G* RNA expression is not sufficient to cause premature SC loss and that the phenotype observed in the *y w; sv*^*spa-pol*^ background is more complex than simply reducing expression of *c(3)G*. In contrast, *y w; sv*^*spa-pol*^ *c(3)G*^*68*^ heterozygotes exhibit relatively normal levels of expression and still exhibit premature SC breakdown in the *y w; sv*^*spa-pol*^ background.

Surprisingly, expression of *c(3)G* RNA in *c(3)G*^*1*^ heterozygotes in all tested backgrounds was less than in *c(3)G*^*68*^ heterozygotes (Fig. S3). Both the *c(3)G*^*1*^ and the *c(3)G*^*68*^ mutations cause premature stop codons, but the large transposon insertion present in the *c(3)G*^*1*^ allele could reduce levels of *c(3)G* transcription or alter the stability of the *c(3)G*^*1*^ transcript. In many systems, transposons have been found to inactivate or alter gene expression when inserted within genes or in nearby regulatory regions (Kazazian 2004; Munoz-Lopez and Garcia-Perez 2010). In *Drosophila*, a particularly nice example of this comes from attempts to identify a causative mutation after a screen. In this case, the gene responsible for the phenotype, *ald*, did not have a mutagenic lesion but instead a *Doc* transposable element in a neighboring gene suppressed the function of *ald* in *cis* in the germline, but not in the soma (Hawley and Gilliland 2006). It is likely that differences in gene expression between *c(3)G*^*1*^ and the *c(3)G*^*68*^ heterozygotes are due to the presence of a transposon in the first exon of *c(3)G* in the *c(3)G*^*1*^ allele.

Because Corolla is also a necessary component of the SC (Collins et al. 2014), *corolla* was sequenced from the ~ 500 bp regions upstream to downstream of *corolla* in each of the control stocks. Only one SNP was present within the coding region of *corolla* in *y w; sv*^*spa-pol*^ when compared to *OreR* or *w*^*1118*^*.* This guanine-to-thymine point mutation would result in an amino acid change from aspartate to glutamate at position 454 in Corolla. Because of the minimal structural difference between aspartate and glutamate, this SNP is likely not significant.

### Premature loss of SC does not disrupt chromosome segregation

To determine if the premature SC breakdown in *y w; sv*^*spa-pol*^ *c(3)G* heterozygotes affected meiotic outcomes, chromosome segregation was assessed. The *y w; sv*^*spa-pol*^ control stock displayed negligible rates of *X* (0.7%) and *4th* (0.3%) chromosome nondisjunction (Table 3: adj. *N* = 585), consistent with previously published results (Hughes et al. 2019; Billmyre et al. 2019). *y w; sv*^*spa-pol*^ *c(3)G*^*68*^ heterozygotes phenocopied these results with nondisjunction rates of 0.0% (*p* = 0.196) and 0.7% (*p* = 0.536) for the *X* and *4th* chromosome, respectively (Table 3: adj. *N* = 409). Nondisjunction rates of the *X* chromosome and the *4th* chromosome were not significantly different between the *y w; sv*^*spa-pol*^ control stock and *y w; sv*^*spa-pol*^ *c(3)G*^*68*^ heterozygotes (Table 3). These rates of nondisjunction differ from the large amount of *X* chromosome (39.2%) and *4th* chromosome (26.8%) missegregation seen in *c(3)G*^*68*^ homozygotes (Hall 1972). The wild-type levels of nondisjunction observed in *y w; sv*^*spa-pol*^ *c(3)G* heterozygotes indicate that premature SC breakdown at stage 2 does not result in the missegregation of chromosomes.

**Table 3:** Premature loss of SC does not impact the rate of *X* and *4th* chromosome nondisjunction in *y w; sv*^*spa-pol*^ and *c(3)G* heterozygous females

Maternal genotype	*y w; sv*^*spa-pol*^	*y w/y; +/+; th c(3)G*^*68*^*/+; sv*^*spa-pol*^*/+*
Adjusted *N* value	585	409
Percent nondisjunction (*p* value)		
*X*	0.7	0.0 (*p* = 0.196)
*4th*	0.3	0.7 (*p* = 0.536)

Calculations performed as described in the methods (Zitron and Hawley 1989; Hawley et al. 1992). Adjusted N accounts for the inviable progeny class plus the scored progeny. Significance calculated as previously described (Zeng et al. 2010)

## Discussion

Here we show that timing of SC breakdown in *c(3)G* heterozygotes is susceptible to the effects of genetic background. Specifically, a *y w; sv*^*spa-pol*^ background significantly accelerated SC breakdown in *c(3)G* heterozygotes compared to *OreR* and *w*^*1118*^ backgrounds (Figs. 2,3). A lesser effect on SC breakdown timing was observed in the *y w; sv*^*spa-pol*^ control stock in the absence of *c(3)G* heterozygosity. By examining SC breakdown in *corolla*^*39*^ heterozygotes in the *y w; sv*^*spa-pol*^ background, we confirmed that the breakdown phenotype is specific to both the *y w; sv*^*spa-pol*^ stock and *y w; sv*^*spa-pol*^ *c(3)G* heterozygotes. Additionally, our analysis of chromosome segregation showed that the premature breakdown of full-length SC early in mid-prophase (stage 2) was not sufficient to cause chromosome missegregation.

### Control backgrounds in *Drosophila*

Many fields have standard control stocks, such as N2 in *Caenorhabditis elegans*, and SK1 and S288c in *S. cerevisiae*. However, in *Drosophila*, multiple control stocks are used depending on the laboratory and the experimental approach. Worse yet, the genetic identity of so-called control stocks can vary from lab to lab. Even if different labs are using stocks with the same known mutations as controls, they may have been maintained separately for decades. Due to the artificial population bottleneck, this can lead to an accumulation of mutations or modifiers; some of which may modify mutant phenotypes. The use of varying control stocks can lead to reproducibility issues between laboratories, thus highlighting the importance of genetic background in experiments.

### Why does the *y w; sv*^*spa-pol*^ background cause premature SC breakdown in *c(3)G* heterozygotes?

The control stock, *y w; sv*^*spa-pol*^, is often used for chromosome segregation studies because of the utility of the phenotypic markers present. The *y* and *w* mutant alleles are used to assay both gain and loss of the *X* chromosome, while the *sv*^*spa-pol*^ allele is used to test for *4th* chromosome nondisjunction. The *y w; sv*^*spa-pol*^ stock used in this study was created in the Hawley lab in 2009 and has been passaged by serial transfer since then, potentially allowing for modifiers to accumulate.

Perhaps one explanation of our data is that this stock carries a hypomorphic mutation in a gene (or several genes) required for the control of SC disassembly during meiosis. Such an explanation would also propose that this mutation can act as a dominant enhancer in *c(3)G* heterozygotes, and the gene product acts in some manner along with the C(3)G protein in a dosage-dependent fashion. Identifying this gene would be interesting as it might encode a protein that serves to maintain the SC. However, uncovering the identity of this potential modifier(s) of SC breakdown would require both extensive mapping studies and meticulous genome sequence analysis, which lies outside the scope of this study.

### Why is there a difference between *c(3)G* and *corolla*?

One of the surprising findings of this study was the presence of a phenotype in *y w; sv*^*spa-pol*^ *c(3)G* heterozygotes but not in *y w; sv*^*spa-pol*^ *corolla*^*39*^ heterozygotes (Fig. 4). As noted above, it is possible that a modifier exists in the *y w; sv*^*spa-pol*^ background that is specific to *c(3)G* and not to the entire SC structure. The presence of a *c(3)G* specific modifier in the *y w; sv*^*spa-pol*^ background is supported by the identical shift to earlier breakdown in both *c(3)G* null mutants. These two mutants carry very different mutations and have been maintained in different backgrounds, so it is unlikely that the background of the *c(3)G* mutants is contributing to their identical phenotype. A *c(3)G* specific modifier would explain the absence of a premature SC breakdown phenotype in *y w; sv*^*spa-pol*^ *corolla*^*39*^ heterozygotes. Assuming that the *y w; sv*^*spa-pol*^ control stock has two alleles of this *c(3)G* modifier, a mild premature SC breakdown phenotype could be anticipated if the modifier was decreasing the amount of C(3)G present. Further, a stronger phenotype would be expected in a heterozygote for a null allele of *c(3)G* in the *y w; sv*^*spa-pol*^ background where even less C(3)G protein might be made, but not in a *corolla* heterozygote where only one copy of the modifier would be present along with two wild-type copies of *c(3)G*. Interestingly, our qPCR data supports a *y w; sv*^*spa-pol*^-specific reduction in *c(3)G* RNA (Fig. S3), but this is not sufficient to explain the premature breakdown phenotype in *y w; sv*^*spa-pol*^ *c(3)G* heterozygotes as the levels of *c(3)G* transcript are not further reduced when compared to the *y w; sv*^*spa-pol*^ background (Fig. S3). Unfortunately, the existing C(3)G antibody is incompatible with western blots, making it difficult to assess C(3)G protein levels. Taken together, our data suggest that while *c(3)G* RNA levels are reduced in a *y w; sv*^*spa-pol*^ background, reduction at the RNA level is insufficient to fully explain the premature SC breakdown phenotype.

### The importance of regulating the disassembly of the SC

Our work here shows that there is extensive variability in the timing of SC breakdown both within stocks and between stocks. However, it is unclear if the premature breakdown defect seen in *y w; sv*^*spa-pol*^ *c(3)G* heterozygotes is due to a loss of SC maintenance resulting in premature SC breakdown or active premature SC disassembly. In *Drosophila,* it is unknown if euchromatic SC disassembly and/or SC maintenance are actively regulated processes or if the SC disassembles passively over time as there is evidence supporting both hypotheses. In some organisms, like *C. elegans*, it is known that there are separate mechanisms that actively maintain the SC and actively disassemble it (reviewed in Gao and Colaiácovo 2018).

In most *Drosophila* stocks, euchromatic SC is completely disassembled by stage 8 or 9, but timing of the beginning of SC breakdown greatly varies, suggesting that SC disassembly at later stages is a passive process. One caveat is that we are limited by ovariole morphology when quantifying the stage of SC disassembly. Not every stage is present in a single ovariole, meaning that the observed stage of SC disassembly may be different than the actual stage when the SC first initiates disassembly. Therefore, analysis of SC disassembly is limited to what stages of development are present at the time of fixation.

Some mutants contain large SC polycomplexes which are complex, SC-like structures appearing to be composed of repeating units of SC proteins (Hughes et al. 2019). These SC polycomplexes can persist until after the stage of normal SC disassembly (Hughes et al. 2019), suggesting there is an active mechanism in wild-type flies that removes the SC by stage 9. Additionally, there are likely different mechanisms of SC breakdown in early versus late meiosis. In early meiosis, multiple cells form SC in the germarium (Fig. 1) prior to a single oocyte being designated. By stage 1, the other cells rapidly disassemble the SC before becoming nurse cells that support the oocyte. There is very little variation in the disassembly of the SC in the future nurse cells, suggesting there must be active removal of the SC at this stage. However, without more information regarding the mechanisms that regulate SC breakdown, it is impossible to know if the processes of maintenance and disassembly are linked or independent events.

In *Drosophila*, the SC must assemble for normal levels of DSBs, and, therefore, COs to occur; also, the SC must be maintained in early prophase to preserve pairing and normal recombination patterns (Page and Hawley 2001; Page et al. 2008; Collins et al. 2014; Billmyre et al. 2019). However, SC assembly is not dependent on the formation of DSBs or recombination in *Drosophila *(McKim and Hayashi-Hagihara 1998). By examining systems where the SC breaks down prematurely, like the one reported here, we can try to dissect why full-length SC is maintained in later stages after DSB initiation and CO specification occur.

Moreover, the observation that early SC breakdown in *y w; sv*^*spa-pol*^ flies does not affect chromosome segregation suggests that full-length SC might be unnecessary after stage 1, which may have allowed variation to arise in the system. This is apparent in the wide range of SC breakdown (stage 1–8) present in the *y w; sv*^*spa-pol*^ control stock, which is larger than the ranges in *OreR* and *w*^*1118*^ (stage 4–9 and stage 6–9, respectively).

Our result showing that SC breakdown along the euchromatin at stage 2/3 caused minimal nondisjunction is consistent with previous work showing that nondisjunction does not occur when the SC breaks down in stage 1 (Billmyre et al. 2019). Thus, full-length SC appears to be dispensable after stage 1 for chromosome segregation. It has been suggested that the SC needs to remain at the centromere until later in meiosis for proper chromosome segregation (Gladstone et al. 2009; Takeo et al. 2011; Qiao et al. 2012; Bisig et al. 2012). The work presented here does not directly address the role of centromeric SC in chromosome segregation. At the stage of disassembly, the majority of our samples contained fragmented or punctate SC, which was likely at the centromeres. However, some oocytes did not display clear SC staining at the stage of disassembly and instead had a diffuse haze, suggesting that in *Drosophila,* Corolla staining is sometimes not detectable along the chromosome arms or at the centromeres in later stages. This is in line with previous reports showing no SC associated with chromosomes in 63% and 89% of stage 8 and stage 9 oocytes in *OreR* flies (Resnick et al. 2009). Further work is needed to better understand the breakdown of euchromatic and centromeric SC and how it influences chromosome segregation in *Drosophila*.

In summary, the work presented here provides insight into how genetic background might impact future studies of *c(3)G* and other meiotic genes. Our results show that not all control backgrounds exhibit identical patterns of SC breakdown and highlights the importance of considering genetic background when analyzing phenotypes, as modifiers or mutations in the control background could lead to false conclusions.

## Materials and methods

### Drosophila stocks

*Drosophila* stocks were maintained on standard media at 24°. Descriptions of genetic markers and chromosomes can be found at http://www.flybase.org/. The control stocks used within these experiments include *y w; +/+; +/+; sv*^*spa-pol*^, *Oregon-R*, and *w*^*1118*^. These stocks are referred to as *y w; sv*^*spa-pol*^, *OreR*, and *w*^*1118*^ respectively. These control stocks have been maintained in the Hawley lab for many years.

The stocks of null alleles of *c(3)G* were: *+/Y; +/+; ru h th st c(3)G*^*1*^ *e*^*s*^ *ca/TM3, Sb Ser; +/+* (referred to as *c(3)G*^*1*^) and *y/y*^*+*^*Y; +/+; th c(3)G*^*68*^*/TM3; +/+* (referred to as *c(3)G*^*68*^). *c(3)G*^*1*^ contains a 412 bp retrotransposon long terminal repeat insertion resulting in an amino acid change Q115L followed by a stop codon (Page and Hawley 2001). *c(3)G*^*68*^ is the result of a premature stop after 77 amino acids (Page and Hawley 2001). The *c(3)G* heterozygotes created from *y w; sv*^*spa-pol*^ were *y w/y; +/+; th c(3)G*^*68*^*/+; pol/+* (referred to as *y w; sv*^*spa-pol*^ *c(3)G*^*68*^ heterozygotes) and *y w/+; +/+; ru h th st c(3)G*^*1*^ *e*^*s*^ *ca/+; pol/+* (referred to as *y w; sv*^*spa-pol*^ *c(3)G*^*1*^ heterozygotes). The *c(3)G* heterozygotes created from *OreR* were *+(OreR)/y; +/+; th c(3)G*^*68*^*/+; +/+* (referred to as *OreR c(3)G*^*68*^ heterozygotes) and *+(OreR)/+; +/+; ru h th st c(3)G*^*1*^ *e*^*s*^ *ca/+; +/+* (referred to as *OreR c(3)G*^*1*^ heterozygotes). The *c(3)G* heterozygotes created from *w*^*1118*^ were *w*^*1118*^*/y; +/+; th c(3)G*^*68*^*/+; +/+* (referred to as *w*^*1118*^ *c(3)G*^*68*^ heterozygotes) and *w*^*1118*^*/+; +/+; ru h th st c(3)G*^*1*^ *e*^*s*^ *ca/+; +/+* (referred to as *w*^*1118*^ *c(3)G*^*1*^ heterozygotes). The stock of the null allele of *corolla* was *y sc w*^*+*^ *cv v corolla*^*39*^/*Y; +/+; +/+; +/+* (referred to as *corolla*^*39*^). The *corolla*^*39*^ heterozygotes created from *y w; sv*^*spa-pol*^ were *y w/y sc w*^*+*^ *cv v corolla*^*39*^*; +/+; +/+; sv*^*spa-pol*^*/+* (referred to as *y w; sv*^*spa-pol*^ *corolla*^*39*^ heterozygotes). Each heterozygous genotype and its abbreviation are described in Table 1. The heterozygous flies analyzed in the experiment were created by mating female virgin flies from the control stocks to heterozygous males possessing one null allele of *c(3)G* maintained with a third chromosome balancer (See Fig. S2 for Cross Scheme).

### Nondisjunction assays

To measure the rate of both *X* and *4th* chromosome nondisjunction, single virgin females of the indicated genotype were mated to multiple *X^Y, In(1)EN, v f B; C(4)RM, ci ey*^*R*^ males. *X* chromosome nondisjunctional offspring from the female are either *yellow* females (diplo-*X* exceptions) or *vermillion*, *forked*, *Bar* males (nullo-*X* exceptions). *Forth* chromosome nondisjunctional offspring from the female are either *sv*^*spa-pol*^ flies (diplo-4 exceptions) or *ci ey*^*R*^ (nullo-4 exceptions). Nondisjunction frequencies are calculated by adding the exceptional progeny classes and dividing by total of all progeny classes. Because the female test parent contained free *X* chromosomes, the number of exceptional-*X* progeny is doubled prior to calculations to correct for the inviability of triplo-*X* and nullo-*X* progeny. Additionally, because the *c(3)G* heterozygotes were heterozygous for *sv*^*spa-pol*^, it was impossible to score for diplo-4-exceptions. Therefore, only nullo-exceptional progeny were scored from the control and *c(3)G* heterozygous females (Zitron and Hawley 1989; Hawley et al. 1992). *y w; sv*^*spa-pol*^ was the only control stock examined because *OreR* and *w*^*1118*^ do not have the correct markers to assay nondisjunction by this method.

### Immunostaining of whole-mount ovaries

Germarium preparation for whole-mount immunofluorescence was performed as previously described (Page and Hawley 2001). Two to 4-day-old females were collected and yeasted overnight at 24 °C. Ovaries were dissected in under 10 min in PBS with 0.1% Tween (PBST). Ovaries were fixed in 200 μL of PBS containing 2% formaldehyde (Ted Pella, Redding, CA) and 0.5% Nonidet P-40 plus 600-μL heptane at room temperature for 20 min. Then the ovaries were washed three times in PBST for 10 min. Ovarioles were teased apart with forceps and the late stage egg chambers were removed before being blocked for 1 h in PBST with 1% bovine serum albumin (BSA) (EMD Chemicals, San Diego, CA). Ovarioles were incubated overnight with primary antibody diluted in PBST at 4° while nutating. The ovarioles were then washed with PBST three times for 20 min. Ovarioles were incubated for 2 h in secondary antibody in PBST and 4′6-diamididino-2-phenylindole (DAPI) (final concentration of 1 μg/ml) was added for the final 10 min. The ovarioles were washed in PBST three times for 15 min and then mounted in ProLong Gold (Life Technologies, Grand Island, NY). The primary antibody used was affinity-purified rabbit anti-Corolla (1:2000). The secondary antibody, Alexa Fluor 488 goat anti-rabbit (Thermo Fisher, A11008), was used at 1:500.

### Imaging and image analysis

All images were acquired on an inverted DeltaVision microscopy system (GE Healthcare) with an Olympus 100× Objective (UPlanSApo 100× NA 1.40), an Olympus 40× Objective (UApo/340 40× 1.35), and a high-resolution CCD camera. Images were deconvolved (DeltaVision). Images were cropped and brightness and contrast were slightly adjusted using ImageJ. Stage of breakdown was defined as the first instance of fragmentation or punctation of the SC in the specified ovariole. If no fragmentation or punctation was observed, the stage at which a diffuse staining of SC was present was considered the stage of breakdown. The oocytes in the stages following the stage of breakdown were not analyzed. Each category of SC breakdown was considered equal during statistical analysis. We identified three categories of the SC breakdown phenotype: fragmented, punctate, and diffuse SC. Fragmentation was defined as when several instances of discontinuation were noted in the tracks of SC. Punctation was defined as when there were no discernible lengths of SC, but instead multiple punctate foci of SC were present. If no fragmentation was observed, the stage at which the SC was only present in a diffuse pattern was considered the stage of breakdown (Fig. S1). Quantification of these categories in the data set is presented in Table S2-S4. Egg chambers of the ovariole were measured and staged as previously described (Spradling 1993).

### Sanger sequencing

The genes *c(3)G* and *corolla* were sequenced in each stock using Sanger sequencing and compared to each other and to the reference sequence data (FB2020_02) available on FlyBase (Thurmond et al. 2018).

### qPCR analysis

Total RNA from ovaries was isolated using the Promega Maxwell RSC Simply RNA Tissue Kit using standard protocol except for increasing the amount of DNase to 10 μL per sample. cDNA was synthesized from total RNA (1 μg) using the Invitrogen SuperScript III First-Strand Synthesis System for RT-PCR using random hexamers. Each genotype was run in triplicate using Quanta Biosciences PerfeCTa SYBR Green FastMix ROX reagent. The *c(3)G* primer set was 5′-AGCGTGAAAAGAACAATGAAATGGC-3′ and 5′-TGCTCTCAGTTCTGGTTGGCCC−3′. The control transcript primer set used was 5′-AGCGCACCAAGCACTTCATC−3′ and 5′-GACGCACTCTGTTGTCGATACC-3′ for rpL32 (FBgn0002626).

#### Data and software availability

Original data underlying this manuscript can be accessed from the Stowers Original Data Repository at http://www.stowers.org/research/publications/libpb-1531.

## Electronic supplementary material

ESM 1 (DOCX 2994 kb)
