# Supplementary material for: Genetic background impacts the timing of synaptonemal complex breakdown in Drosophila melanogaster
Source: Chromosoma. 2020 Oct 17;129(3):243–54. doi: 10.1007/s00412-020-00742-9 (PMC7666587; doi:10.1007/s00412-020-00742-9)
Supplement: Supplementary file 1 — (DOCX 2994 kb) [file 412_2020_742_MOESM1_ESM.docx]

**Supplemental Material**

**
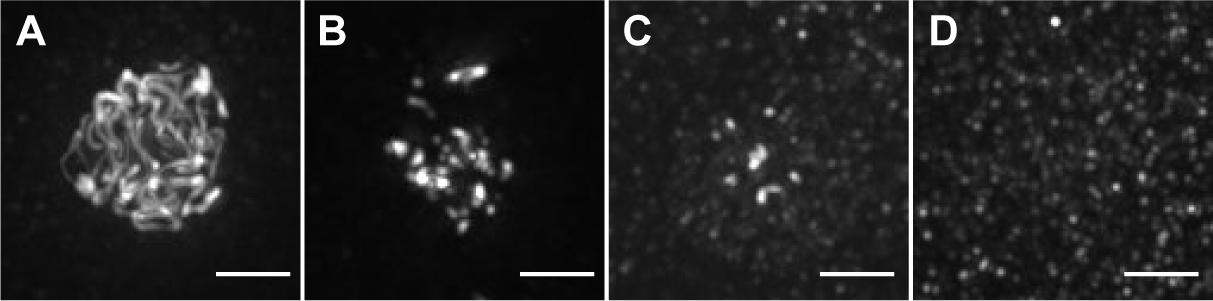
**

**Supplemental Figure 1: Representative images of the four scoring categories.** A) Full-length SC, B) Fragmented SC, C) Punctate SC, and D) Diffuse Corolla staining. (Scale bars, 2 μm) The image of full-length SC is from a stage 3 *w^1118^* ovariole. The image of fragmented SC is from a stage 2 *y w; sv^spa-pol^* *c(3)G^68^* heterozygote ovariole. The punctate image is from a stage 4 *y w; sv^spa-pol^* *c(3)G^68^* heterozygote ovariole. The image of diffuse staining is from a stage 6 *w^1118^* ovariole.

**
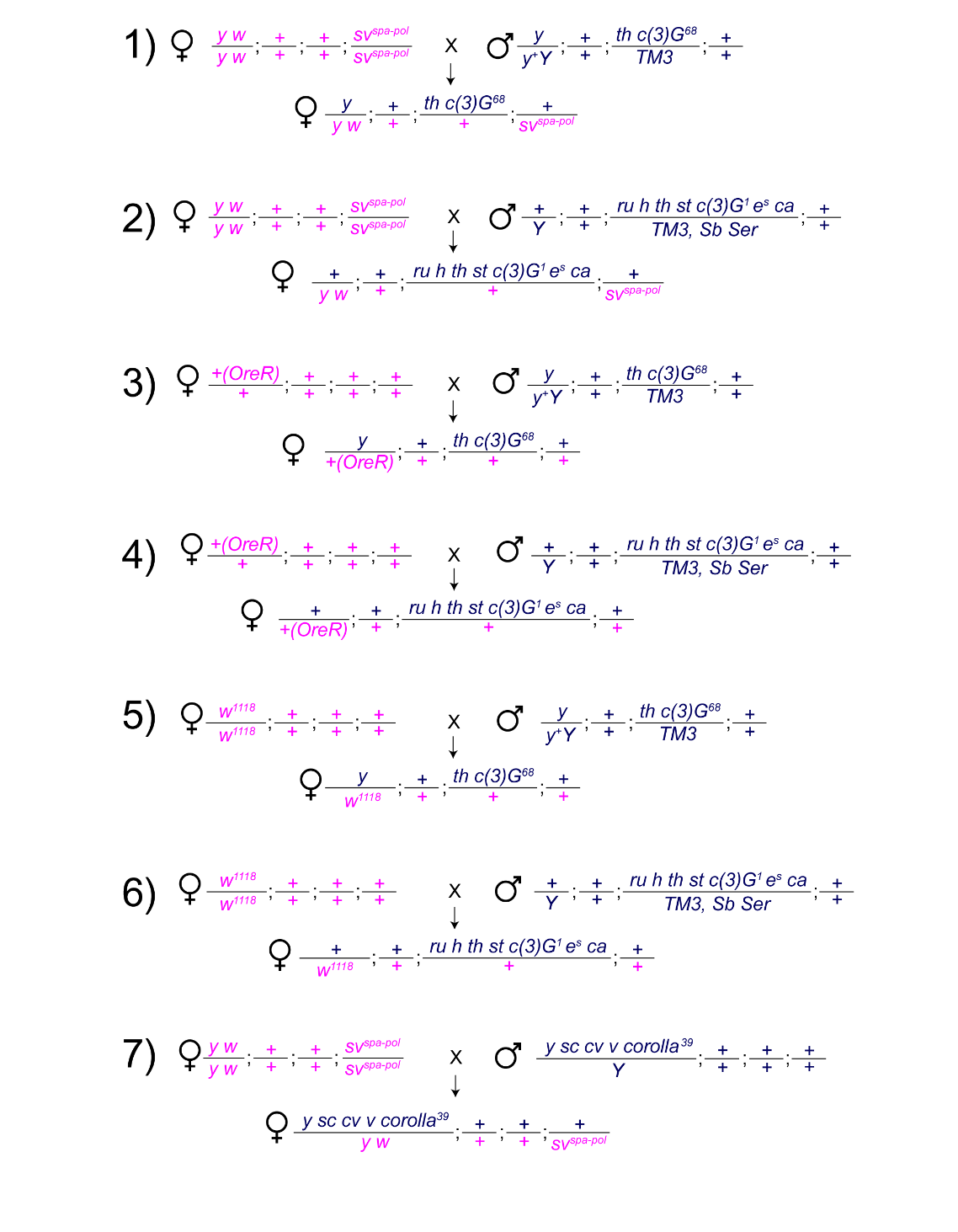
**

**Supplemental Figure 2: Diagram of the cross schemes used to create *c(3)G* and *corolla* heterozygotes with varying control backgrounds.** Maternal genotypes in pink and paternal genotypes in navy. 1) *y w; sv^spa-pol^* *c(3)G^68^* heterozygote, 2) *y w; sv^spa-pol^* *c(3)G^1^* heterozygote, 3) *OreR c(3)G^68^* heterozygote 4) *OreR c(3)G^1^* heterozygote, 5) *w^1118^ c(3)G^68^* heterozygote, 6) *w^1118^ c(3)G^1^* heterozygote, 7) *yw; sv^spa-pol^ corolla* heterozygote. Progeny from crosses 1-6 displayed in Figure 3. Progeny from cross 7 displayed in Figure 4.


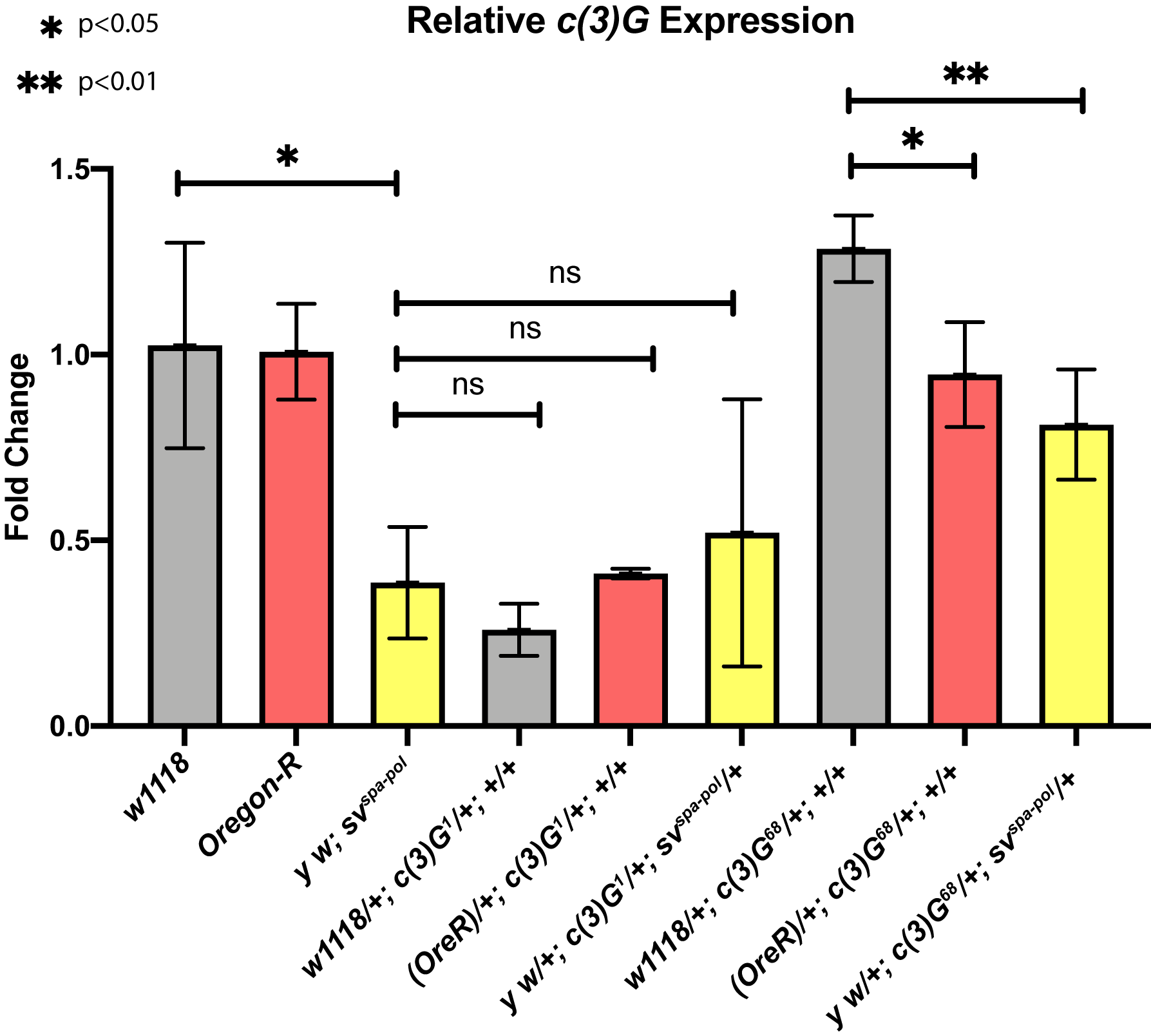


**Supplemental Figure 3: Relative expression of *c(3)G* in ovaries by qPCR.** *rpL32* (a ribosomal protein) was used as an internal control. Samples were normalized to the average expression level of *c(3)G* in *w^1118^* ovaries. Graph is color-coded by the background genotype: *w^1118^* (grey), *Oregon-R* (red), and *y w; sv^spa-pol^* (yellow).

| **Genotype** | **Median stage** | **STDEV** |
| --- | --- | --- |
| *y w; sv^spa-pol^(control for Fig 2)* | 4 | 2.27 |
| *y w; sv^spa-pol^ (control for Fig 4)* | 4 | 2.76 |
| *OreR* | 8 | 1.41 |
| *w^1118^* | 8 | 1.06 |
| *y w/y; +/+; th c(3)G^68^/+; sv^spa-pol^/+* | 2 | 1.83 |
| *y w/+; +/+; ru h th st c(3)G^1^ e^s^ ca/+; sv^spa-pol^/+* | 2 | 1.13 |
| *+(OreR)/y; +/+; th c(3)G^68^/+; +/+* | 7 | 1.44 |
| *+(OreR)/+; +/+; ru h th st c(3)G^1^ e^s^ ca/+; +/+* | 5 | 1.39 |
| *w^1118^/y; +/+; th c(3)G^68^/+; +/+* | 6 | 1.79 |
| *w^1118^/+; +/+; ru h th st c(3)G^1^ e^s^ ca/+; +/+* | 7 | 1.21 |
| *y w/y sc w^+^ cv v corolla^39^; +/+; +/+; sv^spa-pol^/+* | 8 | 1.07 |

**Table S1: Median and standard deviation of stage of SC breakdown for all experimental genotypes.**

| **Genotype** | **Stage** | **Fragmented** | **Punctate** | **Diffuse** |
| --- | --- | --- | --- | --- |
| *y w; sv^spa-pol^  (Fig 2)* | *Stage 1* | 2 | - | - |
| N= 17 | *Stage 2* | 3 | - | - |
|  | *Stage 3* | 2 | - | - |
|  | *Stage 4* | 2 | - | - |
|  | *Stage 5* | 2 | - | - |
|  | *Stage 6* | 1 | - | - |
|  | *Stage 7* | 3 | 1 | - |
|  | *Stage 8* | - | 1 | - |
|  | *Stage 9* | - |  | - |
|  |  |  |  |  |
| *Oregon-R* | *Stage 1* | - | - | - |
| N= 16 | *Stage 2* | - | - | - |
|  | *Stage 3* | - | - | - |
|  | *Stage 4* | 1 | - | - |
|  | *Stage 5* | - | 1 | 1 |
|  | *Stage 6* | 2 | - | - |
|  | *Stage 7* | 1 | - | - |
|  | *Stage 8* | 7 | - | 2 |
|  | *Stage 9* | - | 1 | - |
|  |  |  |  |  |
| *w^1118^* | *Stage 1* | - | - | - |
| N=16 | *Stage 2* | - | - | - |
|  | *Stage 3* | - | - | - |
|  | *Stage 4* | - | - | - |
|  | *Stage 5* | - | - | - |
|  | *Stage 6* | 2 | - | 2 |
|  | *Stage 7* | 2 | 2 | - |
|  | *Stage 8* | - | 3 | 2 |
|  | *Stage 9* | 1 | 1 | 1 |

**Table S2: Raw data for Figure 2**

| **Genotype** | **Stage** | **Fragmented** | | **Punctate** | | **Diffuse** | |  |
| --- | --- | --- | --- | --- | --- | --- | --- | --- |
| *y w/+; c(3)G^68^/+; sv^spa-pol^/+* | *Stage 1* | | 1 | | 2 | | - | |
| N= 17 | *Stage 2* | | 6 | | 1 | | - | |
|  | *Stage 3* | | - | | 1 | | - | |
|  | *Stage 4* | | - | | 1 | | - | |
|  | *Stage 5* | | 1 | | - | | 2 | |
|  | *Stage 6* | | 1 | | - | | - | |
|  | *Stage 7* | | 1 | | - | | - | |
|  | *Stage 8* | | - | | - | | - | |
|  | *Stage 9* | | - | | - | | - | |
|  |  | |  | |  | |  | |
| *y w/+; c(3)G^1^/+; sv^spa-pol^/+* | *Stage 1* | | 4 | | - | | - | |
| N= 17 | *Stage 2* | | 5 | | 3 | | - | |
|  | *Stage 3* | | 1 | | 1 | | - | |
|  | *Stage 4* | | 2 | | - | | - | |
|  | *Stage 5* | | - | | 1 | | - | |
|  | *Stage 6* | | - | | - | | - | |
|  | *Stage 7* | | - | | - | | - | |
|  | *Stage 8* | | - | | - | | - | |
|  | *Stage 9* | | - | | - | | - | |
|  |  | |  | |  | |  | |
| *+(OreR)/+; c(3)G^68^/+; +/+* | *Stage 1* | | - | | - | | - | |
| N=16 | *Stage 2* | | - | | - | | - | |
|  | *Stage 3* | | - | | - | | - | |
|  | *Stage 4* | | 1 | | 1 | | - | |
|  | *Stage 5* | | - | | 1 | | - | |
|  | *Stage 6* | | 2 | | - | | 1 | |
|  | *Stage 7* | | 2 | | 2 | |  | |
|  | *Stage 8* | | 2 | | 3 | | - | |
|  | *Stage 9* | | - | | 1 | | - | |
|  |  | |  | |  | |  | |
| *+(OreR)/+; c(3)G^1^/+; +/+* | *Stage 1* | | - | | - | | - | |
| N=16 | *Stage 2* | | 1 | | - | | - | |
|  | *Stage 3* | | 1 | | - | | - | |
|  | *Stage 4* | | 2 | | 1 | | 3 | |
|  | *Stage 5* | | 1 | | 1 | | 2 | |
|  | *Stage 6* | | - | | - | | 1 | |
|  | *Stage 7* | | - | | 2 | | 1 | |
|  | *Stage 8* | | - | | - | | - | |
|  | *Stage 9* | | - | | - | | - | |
|  |  | |  | |  | |  | |
| *w^1118^/+; c(3)G^68^/+; +/+* | *Stage 1* | | - | | - | | - | |
| N=15 | *Stage 2* | | - | | - | | - | |
|  | *Stage 3* | | 2 | | - | | - | |
|  | *Stage 4* | | 1 | | - | | - | |
|  | *Stage 5* | | 3 | | - | | - | |
|  | *Stage 6* | | 2 | | - | | 1 | |
|  | *Stage 7* | | 2 | | - | | - | |
|  |  | |  | |  | |  | |
| **Genotype** | **Stage** | | **Fragmented** | | **Punctate** | | **Diffuse** | |
| *w^1118^/+; c(3)G^68^/+; +/+*  *(cont.)* | *Stage 8* | | 1 | | 1 | | 1 | |
|  | *Stage 9* | | - | | 1 | | - | |
|  |  | |  | |  | |  | |
| *w^1118^/+; c(3)G^1^/+; +/+* | *Stage 1* | | - | | - | | - | |
| N=17 | *Stage 2* | | - | | - | | - | |
|  | *Stage 3* | | - | | - | | - | |
|  | *Stage 4* | | - | | - | | - | |
|  | *Stage 5* | | 3 | | - | | - | |
|  | *Stage 6* | | 3 | | - | | - | |
|  | *Stage 7* | | 1 | | - | | 3 | |
|  | *Stage 8* | | 2 | | - | | 4 | |
|  | *Stage 9* | | 1 | | - | | - | |
|  |  | |  | |  | |  | |

**Table S3: Raw data for Figure 3**

| **Genotype** | **Stage** | **Fragmented** | **Punctate** | **Diffuse** |
| --- | --- | --- | --- | --- |
| *y w; sv^spa-pol^  (Fig 4)* | *Stage 1* | - | 1 | - |
| N= 10 | *Stage 2* | 2 | 1 | - |
|  | *Stage 3* | - | - | - |
|  | *Stage 4* | 2 | - | - |
|  | *Stage 5* | - | - | - |
|  | *Stage 6* | - | - | - |
|  | *Stage 7* | 2 | - | - |
|  | *Stage 8* | 1 | - | - |
|  | *Stage 9* | - | 1 | - |
|  |  |  |  |  |
| *y w/y sc w+ cv v corolla^39^; sv^spa-pol^/+* | *Stage 1* | - | - | - |
| N= 18 | *Stage 2* | - | - | - |
|  | *Stage 3* | - | - | - |
|  | *Stage 4* | - | - | - |
|  | *Stage 5* | - | - | - |
|  | *Stage 6* | 2 | - | - |
|  | *Stage 7* | 6 | - | - |
|  | *Stage 8* | 3 | - | - |
|  | *Stage 9* | - | 7 | - |

**Table S4: Raw data for Figure 4**

**Key Resource Table**

| ­REAGENT or RESOURCE | SOURCE | IDENTIFIER |
| --- | --- | --- |
| **Antibodies** | | |
| Corolla rabbit | Hawley Lab | AP-Corolla |
| Alexa Fluor 488 goat anti-rabbit | ThermoFisher | A11008 |
| **Chemicals and Reagents** | | |
| Nonidet-P40 | Sigma-Aldrich | 11332473001 |
| 16% Formaldehyde | Electron Microscopy Sciences | 15710 |
| Prolong Gold | Life Technologies | P36930 |
| **Deposited Data** | | |
| Stowers Original Data Repository (ODR) | Stowers Institute for Medical Research | <http://www.stowers.org/research/publications/odr> |
| Stowers ImageJ Custom Plugins | Stowers Institute for Medical Research | [research.stowers.org/imagejplugins/zipped_plugins.html](http://research.stowers.org/imagejplugins/zipped_plugins.html) |
| **Experimental Model: Drosophila stocks used** | | |
| *y w; +/+; +/+; sv^spa-pol^* | Hawley Lab | Control stock |
| *+(OreR)/+; +/+; +/+; +/+* | Hawley Lab | Control stock |
| *w^1118^/+; +/+; +/+; +/+* | Hawley Lab | Control stock |
| *y w/y; +/+; th c(3)G^68^/+; pol/+* | Hawley Lab |  |
| *y w/+; +/+; ru h th st c(3)G^1^ e^s^ ca/+; pol/+* | Hawley Lab |  |
| *+(OreR)/y; +/+; th c(3)G^68^/+; +/+* | Hawley Lab |  |
| *+(OreR)/+; +/+; ru h th st c(3)G^1^ e^s^ ca/+; +/+* | Hawley Lab |  |
| *w^1118^/y; +/+; th c(3)G^68^/+; +/+* | Hawley Lab |  |
| *w^1118^/+; +/+; ru h th st c(3)G^1^ e^s^ ca/+; +/+* | Hawley Lab |  |
| *y sc w^+^ cv v corolla^39^/Y; +/+; +/+; +/+* | Hawley Lab |  |
| *y w/y sc w^+^ cv v corolla^39^; +/+; +/+; sv^spa-pol^/+* | Hawley Lab |  |
| *+/Y; +/+;* *ru h th st c(3)G^1^ e^s^ ca/TM3, Sb Ser; +/+* | Hawley Lab |  |
| *y/y^+^Y; +/+; th c(3)G^68^/TM3; +/+* | Hawley Lab |  |
| **Oligonucleotides** | | |
| caccgcgaattactggacag | Ordered from IDT | Forward primer for amplifying *corolla* |
| ccttctgttccagctgcttg | Ordered from IDT | Reverse primer for amplifying *corolla* |
| caccgcgaattactggacag | Ordered from IDT | Sense primer for sequencing *corolla* |
| ccgccgaaaatcagctgtaa | Ordered from IDT | Antisense primer for sequencing *corolla* |
| gcaaaattggccaaaagatacca | Ordered from IDT | Sense primer for sequencing *corolla* |
| ggccctacaactgaacaagc | Ordered from IDT | Antisense primer for sequencing *corolla* |
| acggaatccatttgaatgct | Ordered from IDT | Sense primer for sequencing *corolla* |
| taattgtccactcgcgatcc | Ordered from IDT | Antisense primer for sequencing *corolla* |
| ccatgacgagcgtgaataga | Ordered from IDT | Sense primer for sequencing *corolla* |
| agccaatcggtcttctcaaa | Ordered from IDT | Antisense primer for sequencing *corolla* |
| ccaatgaaatttccgataaacc | Ordered from IDT | Sense primer for sequencing *corolla* |
| tcatcgcgattgctaccata | Ordered from IDT | Antisense primer for sequencing *corolla* |
| ttgccatttgcaatcaaaaa | Ordered from IDT | Sense primer for sequencing *corolla* |
| aagcaacgacaaaatgaagaa | Ordered from IDT | Antisense primer for sequencing *corolla* |
| aataaatggctcggaaacga | Ordered from IDT | Sense primer for sequencing *corolla* |
| tctgttccagctgcttgagg | Ordered from IDT | Antisense primer for sequencing *corolla* |
| gatgcctgcgaagtgtacg | Ordered from IDT | Forward primer for amplifying *c(3)G* |
| tacacaacacacacacagcg | Ordered from IDT | Reverse primer for amplifying *c(3)G* |
| tgcgaagtgtacgagagtcg | Ordered from IDT | Sense primer for sequencing *c(3)G* |
| agattcctccattgccttcc | Ordered from IDT | Antisense primer for sequencing *c(3)G* |
| gcttgccagtttcagacaagt | Ordered from IDT | Sense primer for sequencing *c(3)G* |
| ctctttaagcggcaaacgtg | Ordered from IDT | Antisense primer for sequencing *c(3)G* |
| gcacagccatttcattctgtt | Ordered from IDT | Sense primer for sequencing *c(3)G* |
| gcgaggaattacagtctcagaaa | Ordered from IDT | Antisense primer for sequencing *c(3)G* |
| ttcttgaatttcattaccaagacg | Ordered from IDT | Sense primer for sequencing *c(3)G* |
| tctccttgacagattgttgttca | Ordered from IDT | Antisense primer for sequencing *c(3)G* |
| cacgttgcagtttcgacaaa | Ordered from IDT | Sense primer for sequencing *c(3)G* |
| caaagagctccacgaagacc | Ordered from IDT | Antisense primer for sequencing *c(3)G* |
| gctcgcagtgggtgtacttt | Ordered from IDT | Sense primer for sequencing *c(3)G* |
| catggactcgctgaagaaaa | Ordered from IDT | Antisense primer for sequencing *c(3)G* |
| tggcttttcgtttgatctttc | Ordered from IDT | Sense primer for sequencing *c(3)G* |
| aattagttgcgctaatacaactgc | Ordered from IDT | Antisense primer for sequencing *c(3)G* |
| tacataatggcgggatctgg | Ordered from IDT | Sense primer for sequencing *c(3)G* |
| cgtgtgctaagccaaaaatg | Ordered from IDT | Antisense primer for sequencing *c(3)G* |
| ccatccaattccgaaacaaa | Ordered from IDT | Sense primer for sequencing *c(3)G* |
| cgcactttcgactagcgact | Ordered from IDT | Antisense primer for sequencing *c(3)G* |
| gagtcacgccattgtgtttg | Ordered from IDT | Sense primer for sequencing *c(3)G* |
| aggttttcagcgcgaaagta | Ordered from IDT | Antisense primer for sequencing *c(3)G* |
| ccgcaatgatttcgtcagta | Ordered from IDT | Sense primer for sequencing *c(3)G* |
| gctttggccaacaagagaac | Ordered from IDT | Antisense primer for sequencing *c(3)G* |
| **Software and Algorithms** | | |
| ImageJ | <https://imagej.nih.gov/ij/> |  |
| Custom ImageJ plugins | [research.stowers.org/imagejplugins/zipped_plugins.html](http://research.stowers.org/imagejplugins/zipped_plugins.html) |  |
